# The Novel Design of a Single-Sided MRI Probe for Assessing Burn Depth

**DOI:** 10.3390/s17030526

**Published:** 2017-03-06

**Authors:** Zhonghua He, Wei He, Jiamin Wu, Zheng Xu

**Affiliations:** State Key Laboratory of Power Transmission Equipment and System Security and New Technology, School of Electrical Engineering, Chongqing University, Chongqing 400044, China; hewei@cqu.edu.cn (W.H.); wujiamin@cqu.edu.cn (J.W.); xuzheng@cqu.edu.cn (Z.X.)

**Keywords:** unilateral magnetic resonance imaging (UMRI), burn depth assessment, planar gradient coil

## Abstract

Burn depth assessment in clinics is still inaccurate because of the lack of feasible and practical testing devices and methods. Therefore, this process often depends on subjective judgment of burn surgeons. In this study, a new unilateral magnetic resonance imaging (UMRI) sensor equipped with a 2D gradient coil system was established, and we attempted to assess burns using unilateral nuclear magnetic resonance devices. A reduced Halbach magnet was utilized to generate a magnetic field that was relatively homogeneous on a target plane with a suitable field of view for 2D spatial localization. A uniplanar gradient coil system was designed by utilizing the mainstream target field method, and a uniplanar RF (radio frequency) coil was designed by using a time-harmonic inverse method for the UMRI sensor. A 2D image of the cross sections of a simple burn model was obtained by a fast 2D pure-phase encoding imaging method. The design details of the novel single-sided MRI probe and imaging tests are also presented.

## 1. Introduction

Burn depth measurement is crucial in the clinical management of burned patients. However, burn depth assessment in clinics is still inaccurate because of the lack of feasible and practical testing devices and methods. Therefore, this process often depends on a subjective judgment of burn surgeons [1,2,3]. Various approaches have been employed to provide an objective assessment of burn depth. These approaches include biopsy and histology, optical measurement, ultrasound, high-field nuclear magnetic resonance imaging (MRI), and different perfusion measurement techniques, including laser Doppler technique, thermography, and video microscopy [1,2]. However, few of these techniques have become widely adopted in clinics. Although subjective judgment is only accurate in 60%–75% of cases, even for an experienced burn surgeon, clinical assessment remains the most commonly applied method for burn depth assessment [3].

Unilateral nuclear magnetic resonance (NMR) is an effective, non-invasive technique that has been widely utilized in various applications, such as well logging and nondestructive testing of human skin [4,5,6]. This technique can also provide skin characterization, such as relaxation times, self-diffusion coefficients, and relaxation–diffusion [6]. A noninvasive open tomograph can be achieved when a single-sided probe is equipped with a 2D gradient coil system to obtain spatial resolution [7,8]. Although the magnetic field generated by a unilateral magnet is strongly inhomogeneous, considerable effort has been exerted to develop unilateral MRI (UMRI) systems for 2D imaging [7,8,9,10]. Different approaches have been utilized to improve the uniformity of magnetic field generated by the unilateral magnets. These approaches include the horseshoe-type or U-type design that produces transverse polarizing fields [8,11], designs of simple rectilinear or cylindrical bar magnets that produce longitudinal fields [7,12], complex arrangements of magnets [13], and implementation of field-shaping or shimming elements [8,14,15]. In the current work, we presented a new reduced Halbach magnet, instead of the classical U-shaped geometry, to generate a magnetic field that is relatively homogeneous on a target plane. The optimization procedure is simple and could minimize the trial-and-error procedure time to design a single-sided magnet. In addition to the magnet design, any MRI system must include an RF coil that generates a field with components perpendicular to the *B*_0_ field and the gradient coils that produce fields with components parallel to the *B*_0_ field. These components vary linearly as a function of position. The design of planar RF and gradient coil is difficult and poses a significant challenge to single-sided MRI devices for various reasons, such as coil geometry, efficiency, coil size, and sensitivity. For example, the design of planar gradient coils is a challenge primarily because of the difficulty in generating gradient fields that are linear and maximally uniform with a planar coil design. Although Blumich [8,9] presented a gradient coil system utilizing two solenoids wound in an antiparallel configuration and two rectangular coils wound in a parallel configuration for a U-type magnet NMR, four rectangular coils in parallel configuration were also positioned and driven in pairs for a bar magnet NMR. Nevertheless, the challenge of designing planar gradient coils still exists. For planar coil design in transverse single-sided imaging systems, a surface gradient coil design presented by Cho [16] produces a field that is suitable for *x*- and *y*-gradient coils, with transverse components that vary linearly as a function of position. Viktor Vegh [17] also presented *x*- and *y*-gradient planar coil designs with a path correction method suitable for UMRI systems. However, the mainstream target field approach [18,19] has not been considered to design planar gradient coils for a UMRI system. The most common design of RF coils explored for UMRI sensors is a single current loop in the plane for a *B*_1_ field perpendicular to the face of the coil and a figure-of-eight-like coil with two opposing current loops for a *B*_1_ field parallel to the face of the coil [4]. Despite several special RF coils, such as microstructured [20] and arc-shaped spiral RF coils [21], adopted for a signal-to-noise ratio (SNR) and sensitivity improvement, the RF coil design considerations for UMRI systems have been largely overshadowed by the attention given to single-sided magnet design. RF coil efficiency is critical at low fields typical of single-sided systems. The time-harmonic target field method has been used to design various RF coils with high magnetic field homogeneity for imaging of breast and head [22,23], but it has not been applied to design RF coils for UMRI systems. Therefore, we consider the design of a planar RF coil utilizing the time-harmonic target field approach to obtain a high sensitivity and SNR. The design of gradient coils utilizing the target field method is also considered to maximize the degree of linearity and minimize power consumption for the UMRI system.

In the current work, we propose a new UMRI system for assessing burns. This system is composed of an optimal single-sided magnet utilizing a reduced Halbach magnet, uniplanar gradient coils designed using the mainstream target field method, and an RF coil designed utilizing the time-harmonic inverse design. Thus, providing a small, economical UMRI device capable of performing various studies on arbitrarily large surfaces, such as burn wound skin, would be advantageous. A prototype UNMR system was designed and constructed, and the 2D imaging results of a simple burn model are presented in this paper.

## 2. Sensor Design

The new low-field UMRI probe for 2D imaging consisted of three main parts: a magnet system, a gradient coil, and an RF coil.

### 2.1. Magnet System

**Figure 1:**
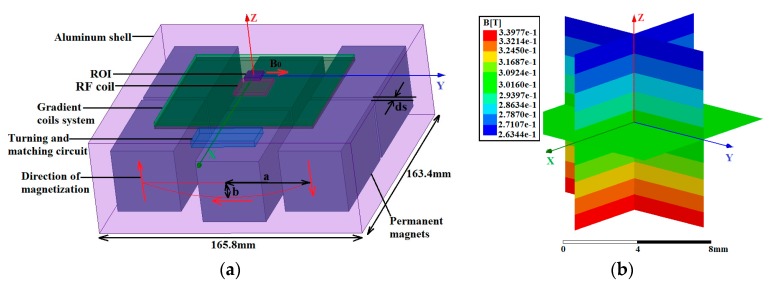
(**a**) Newly reduced Halbach magnet equipped with a 2D gradient coil system. This magnet was created by utilizing six NdFeB magnet blocks. The polarization directions of the magnets and main field are indicated by red arrows. The six magnets are polarized along the −*z*-, −*y*-, and +*z*-directions. The magnets pointing in the same polarization direction were separated by a small gap ds, whereas the magnets pointing to different polarization directions formed a reduced Halbach structure and were positioned in an elliptical arc. The ratio of the semimajor axis *a* to the semiminor axis *b* was 2.3. (**b**) Simulation results of the optimized reduced Halbach magnet structure. The magnetic field was computed using the Ansoft finite element method (FEM) software. The calculated magnetic field is shown for distances to the center of the six-magnet array lower than 5 mm and above the magnet array surface at 10–20 mm. At the target plane, the simulated magnetic magnitude was about 0.299 T points along *y*, and the constant gradient was approximately 7.6 T/m points along *z*. A homogeneous magnetic field was produced on the horizontal target plane.

We acquired our magnet design idea from the Halbach and Mandhala arrays [24]. The Halbach array was composed of only four cuboid magnets, and the magnet element on top of the structure was removed as a single-sided structure. The remaining three cuboid magnets were divided into six cuboid magnets with a gap ds. The reduced version of Halbach magnet structure is shown in Figure 1a. The main *B*_0_ field was established by utilizing six NdFeB magnet blocks, and the size of each magnet was 40 mm × 40 mm × 75 mm. The polarization directions of the magnets and the main field are indicated by red arrows. These magnets were polarized along the −*z*-, −*y*-, and +*z*-directions. Magnets pointing to the same polarization direction were separated by a small gap ds, whereas magnets pointing in different polarization directions formed a reduced Halbach structure and were positioned in an elliptical arc. The region of interest (ROI) size was 10 mm × 10 mm × 0.2 mm, and the target plane was positioned at the middle height of the ROI. A 15 mm distance existed between the target plane and the upper surface of the six-magnet array. This distance was reserved for the gradient and RF coils.

Two optimization steps are available for the magnet system. First, the ratio of the semimajor axis *a* to the semi-minor axis *b* was properly selected. During the optimal procedure, *a/b* was changed from 0.5 to 3.5, and the variations in the *B*_0_ strength along the two orthogonal central lines on the target plane were calculated for 31 different structures. The simulations were calculated using the finite element method (FEM) program, which was coded by our team utilizing the Matlab language. In this work, for *a/b* = 1, the *B*_0_ field was shaped similar to an arms-down parabola along the *y*-axis. For *a/b* = 2.3, the magnetic magnitude was almost constant in an area with a radius less than 5 mm on the target plane. When the ratio further increased to 3, the *B*_0_ profile generated an arms-up parabola along the *y*-axis, which showed that an optimum ratio of *a/b* existed for a constant field along *y*. The optimal procedure was similar to that in [25].

Further optimization with a gap ds along *x* was also applied. The solution that introduced a gap ds along *x* was useful in compensating for the end-effect of limit-long magnets [26]. For ds = 0 (three magnets of initial configuration), a strong variation in magnetic field existed in the *x*-direction. For ds = 3.6 mm, the magnetic field magnitude remained constant in an area with a radius of less than 5 mm on the target plane. When the gap further increased to 6.4 mm, the curvature of the magnetic field *B*_0_ was reversed, which showed that an optimum ds existed for a constant field in the *x*-direction. Therefore, a relatively uniform ROI was obtained when the ratio *a/b* and gap ds were properly selected.

To validate the feasibility of design, the magnetic field of the optimized magnet structure was computed using the Ansoft FEM software (ANSYS, Pittsburgh, PA, USA). The calculated magnetic field for distances to the center of the six-magnet array lower than 5 mm and above the magnet array surface at 10–20 mm is shown in Figure 1b. The constant gradient was approximately 7.6 T/m points along *z*, and the magnetic field was homogeneous in a horizontal slice at a certain distance above the sensor. The uniformity reached 234 ppm on the target plane within the ROI, and the magnitude was about 0.299 T.

**Figure 2:**
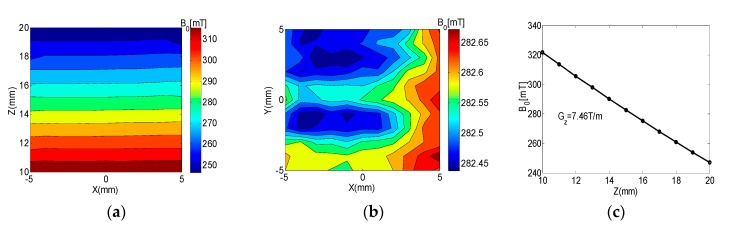
Measured results of the optimized magnet structure. (**a**) Measured field on the *xoz* plane; (**b**) The magnitude of the measured field on the *xoy* target plane was approximately 0.282 T; (**c**) Actual constant gradient was about 7.46 T/m points along *z*.

The optimized magnet was implemented, and the measured magnetic field strength at the *xoz* and *xoy* planes are shown in Figure 2. The measured field at the *yoz* plane was similar to the field at the *xoz* plane and is not shown because of space limitations. A step motor (42BYG250cll, CH-Hall, Beijing, China) and a Bell Gauss/Tesla Meter (Model 7010, F.W.Bell (OECO), Milwaukie, OR, USA) with a resolution of 0.1 mG were employed for measurement. At the target plane, the magnetic field pointed along *y*, the magnitude of the measured field on the *xoy* target plane was approximately 0.282 T, and the actual constant gradient was about 7.46 T/m points along *z*. The most uniform measured plane was located at 14.9 mm away from the surface of the magnet.

### 2.2. Uniplanar Gradient Coils

The gradient coil design idea was obtained from simple geometries of surface gradient coil in [16]. In this reference, the subsections at each *y*-level of *x*-gradient coils are symmetrical and present opposing current polarity with respect to the *y*-axis. The subsections are also symmetrical and show the same current polarity with respect to the *x*-axis. Assuming that the surface current density of gradient coil is restrained on the *x*o*y* plane $\left(-Lx\leq x\leq Lx,-Ly\leq y\leq Ly,z=z_{s}\right)$ and that a set of current-carrying wire arrays on the plane of the gradient coil is present similar to the wire array, the *x* component of the surface current density can be expressed as a Fourier series in 2D according to the current symmetry [27]. Subsequently, the stream function $\psi_{z}(x,y)$ and *y* component of the surface current density $J_{y}(x,y)$ can be obtained according to the current continuity equation. Finally, the appropriate coil winding locations corresponding to the current density in discrete form are obtained using the stream function method. For *y*-gradient coils, the Fourier series for expressing surface current density can be similarly obtained.

**Figure 3:**
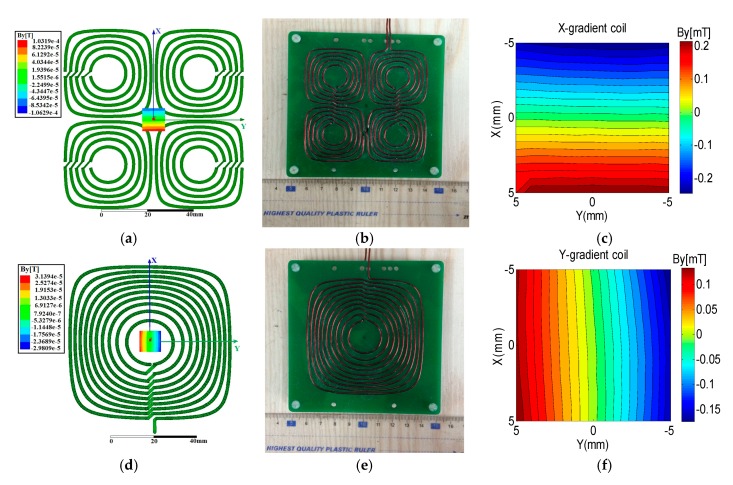
(**a**) Coil winding and gradient field simulated in the Maxwell 3D software for *x*-gradient coil; (**b**) Practical *x*-gradient coil; (**c**) Measured gradient field of the *x*-gradient coil; (**d**) Coil winding and gradient field simulated in the Maxwell 3D software for the *y*-gradient coil; (**e**) Practical *y*-gradient coil; (**f**) Measured gradient field of the *y*-gradient coil. Gradient field was linear for *x*- and *y*-gradient coils.

A standard current density solution of the minimum dissipated power utilizing the target field method was presented in detail in [27]. In the present study, the design details for gradient coils are not shown because of space limitations. The geometry of both the *x*- and *y*-gradient coils were as follows: length, 2Ly=100 mm; width, 2Lx=100 mm. The gradient of the two gradient coils was 40 mT/m when the *x*- and *y*-gradient coils were 7.0 and 5.0 mm below the target plane, respectively. The current density coefficients and coil winding locations were solved in Matlab (MathWorks, Natick, MA, USA), and the coil winding patterns were imported to the Ansoft FEM software to verify the gradient field (Figure 3a,d). The current was 1 A. Considering the use of copper wire with a 1 mm diameter, the smallest possible resistance and inductor, and the strongest possible gradient magnetic field, the *x*-gradient coil with five turns in each quadrant was the most optimal configuration. The wires in the four quadrants were connected in series. For the *y*-gradient coil, a total of 10 turns was the most optimal configuration.

The planar *x*- and *y*-gradient coils were implemented using a 1 mm diameter copper wire, as shown in Figure 3b,e. The resistance values of *x*- and *y*-gradient coils were 3.8 and 3.4 Ω. The gradient field was measured by a highly precise constant current source (Model 6654A, Agilent, Santa Clara, CA, USA) and a Bell Gauss/Tesla Meter (Model 7010, F.W.Bell (OECO), Milwaukie, OR, USA). Figure 3c,f shows the practical gradient field *B_y_* for *x*- and *y*-gradient coils on the target plane within the ROI. Figure 3c,f verifies that the generating gradient fields were linear for *x*- and *y*-gradient coils, respectively. The current of the constant current source was 5 A. Therefore, the gradients calculated for *x*- and *y*-gradient coils were 9.6 and 6.7 mT/(m·A), respectively. Two power amplifiers (Model 7224, AE Techron, Elkhart, IN, USA) were employed to drive the gradient coils. The maximum voltage and maximum current of the power amplifiers were 158 V and 50 A, respectively. The rise time of these coils was measured at approximately 50 μs.

### 2.3. Uniplanar RF Coil

**Figure 4:**
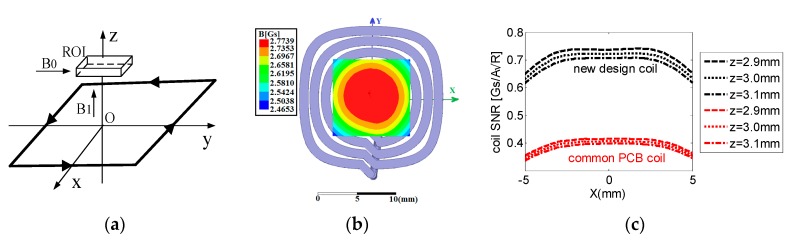
(**a**) Structure of the uniplanar RF coil for unilateral magnetic resonance imaging (UMRI) sensor. (**b**) A solution of the current wire pattern of the coil and simulation result. The simulation is performed using Maxwell 3D software for the UMRI sensor. The central plane of ROI is 3 mm above the current source plane. (**c**) Simulation results of coil SNR. Comparison of the coil SNR at *y* = 0 in the upper, central, and lower planes of the ROI between the designed and common PCB coils.

To improve the sensitivity and SNR of the UMRI probe, the time-harmonic target field method was used to design the RF coil for it. We considered both the basic and specific requirements to produce a *B*_1_ field perpendicular to *B*_0_. A specific requirement is the implementation of *B*_1_ – *B*_0_ matching techniques in the inhomogeneous *B*_0_ field with a constant linear static gradient generated by the unilateral magnet. Therefore, the spins at every field point of each plane in the ROI is flipped to the transverse plane of the field vector simultaneously, and highly resolved correlation spectra and high SNR can be obtained in matched field gradients. The current was symmetrical and presented a closed current loop, as shown in Figure 4a. The direction indicated by the arrow is that of a flowing current, a static main magnetic field, and an RF magnetic field.

The Fourier series to express the current density is the same as that of the *y*-gradient coils, but the magnetic field of each space field point in the ROI is different and must be written as the time-harmonic magnetic vector potential [22]. The coil was located on the plane at 3 mm below the target plane and occupied a region of length 20 mm and width 20 mm. Considering the use of a copper wire with a 1 mm diameter, the smallest possible resistance and inductor, and the strongest possible RF magnetic field, the RF coil with four turns was the most optimal configuration as shown in Figure 4b.

To validate the results of the design, an RF magnetic field was calculated in terms of the current wire patterns of coils by utilizing the Ansoft FEM software. The current was 1 A, and the eddy current solution type was used for simulation. The simulated RF magnetic field was consistent with the design requirement, as shown in Figure 4b. A relatively uniform RF field with a radius less than 5 mm on the target plane was generated.

The poor SNR is the most remarkable challenge among single-sided NMR sensors. Therefore, each new technique utilized for single-sided devices is often evaluated in terms of its sensitivity. The relative SNR $\propto \frac{B_{1}}{i\cdot \sqrt{R}}$ [20] was used in this study to evaluate the designed RF coil, where $B_{1}/i$ is the coil sensitivity that is defined by the law of reciprocity as the magnetic flux density *B*_1_ caused by an RF current i passes through the coil, and R is the alternating-current impedance resistance of the coil. A common printed circuit board (PCB) coil with the same size as the designed RF coil was available for comparison. The common PCB coil was biplanar with 10 turns, and the designed coil was uniplanar with four turns. The simulated R values of the designed and PCB coils were 0.02 and 0.57 Ω, and their *B*_1_ values on the target plane within the ROI were 2.76 and 6.82 Gs, respectively. Hence, the simulation results indicated an averaged SNR improvement with a ratio of approximately 2 as shown in Figure 4c.

## 3. Results and Discussions

**Figure 5:**
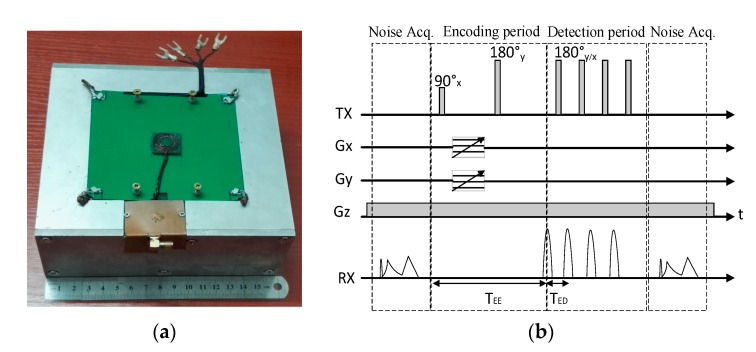
(**a**) New UMRI sensor created with a 2D gradient coil system. The probe measures 166 mm × 164 mm × 69 mm and weighs slightly over 8 kg; (**b**) CPMG-CP sequence utilized for the 2D image. For 1D image, G_y_ was set to zero when the object structures in the *x*-direction was imaged, and G_x_ was set to zero when the object structures in the *y*-direction was imaged. For the 2D image, both G_y_ and G_x_ were set with the gradient amplitude from negative to positive values.

**Figure 6:**
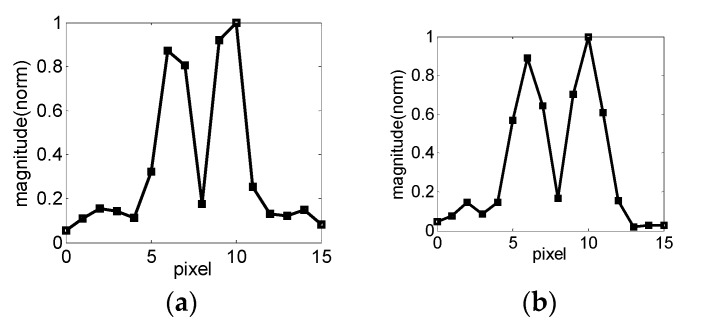
1D images obtained in the *x*- and *y*-directions. A simple object made up of two natural rubber strips was imaged. The rubber strips were 2.5 mm wide and separated by 3 mm. The *k* space was sampled with the gradient amplitude increasing from negative to positive values, and the total steps were 11. The *k* space was zero filled up to 16 points before Fourier transformation. (**a**) 1D image obtained after orienting the object structure in the *x*-direction. Approximately 10 mm field of view (FoV) was obtained using a gradient pulse length of 270 μs, maximum amplifier current of 4.7 A, and G_x,max_= 45 mT/m. (**b**) 1D image of the object structure in the *y*-direction. Approximately 10 mm FoV was obtained using a gradient pulse length of 270 μs, a maximum amplifier current of 6.7 A, and a G_y,max_ of 45 mT/m.

The new UMRI sensor created with a 2D gradient coil system is shown in Figure 5a. The probe measures 166 mm × 164 mm × 69 mm and weighs slightly over 8 kg. The uniplanar RF coil was connected to a Redstone spectrometer (Tecmag, Houston, TX, USA) through a tuning and matching circuit. A Tomco amplifier model BT00500 Alpha-sa (Tomco, Stepney, Australia) was used as the RF power amplifier, and two Techron amplifiers model 7224 were utilized as gradient power amplifier. 1D images were observed in the *x*- and *y*-directions to evaluate the space encoding. An object geometry made up of two strips of natural rubber was utilized to evaluate possible distortions in each direction. The natural rubber strips measured 2.5 mm × 6 mm × 4 mm and were separated by 3 mm. The excitation frequency of the probe was tuned at 12.0 MHz, which produced selective excitation of thin flat slices at a 2.9 mm depth from the RF coil surface. The longitudinal relaxation time *T*_1_ of the natural rubber was approximately 20 ms, but a recycling delay of 100 ms was selected by considering the vibration of the gradient coils. The sequence was a part of the Carr-Purcell-Meiboom-Gill (CPMG)-Carr-Purcell (CP) sequence [4] (Figure 5b) for 1D imaging. The 1D imaging method is similar to the method proposed by Prado [28], but the gradient pulse was switched after the first RF pulse and dozens of 180° pulse were followed to obtain numerous echoes co-added for SNR improvement. When the object structure in the *x*-direction was imaged, G_y_ was set to zero, and the object structure in the *y*-direction was imaged, G_x_ was also set to zero, and only the CPMG sequence was needed. The encoded echo time and detection echo time (T_ED_) were 0.386 and 0.110 ms, respectively. The *k* space was sampled when gradient amplitude increased from negative to positive values, and the total steps were 11. Given that the maximum 50 A driving current could sufficiently drive the gradient coils, the encoding times *t_x_* and *t_y_* in each case were kept constant at 270 μs. A suitable field of view (FoV) was selected by adjusting the maximum current of the gradient amplifier, and 128 scans were used for SNR improvement. A 1D image in the *x*-direction is shown in Figure 6a, and the maximum current of the amplifier was 4.7 A. The total time to obtain the 1D image was approximately 141 s, and the spatial resolution was 1 mm. The same object, but with the structure aligned in the *y*-direction, is shown in Figure 6b. The maximum current of the amplifier was adjusted to 6.7 A because of the smaller value of G_y_ than that of G_x_. Figure 6 shows a similar quality in the *x*- and *y*-directions, and the symmetry of the FoV can be observed. Therefore, this design is suitable for the 2D image reconstruction.

**Figure 7:**
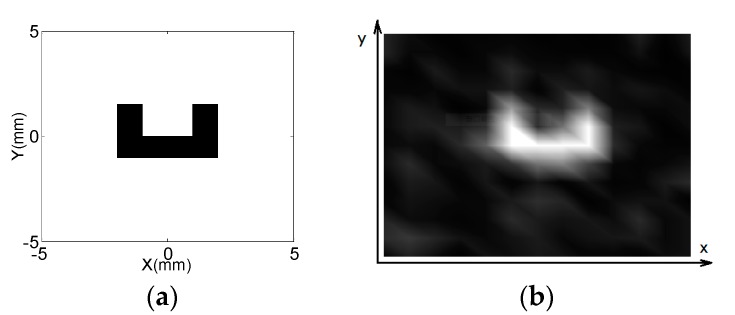
(**a**) Letter C image cropped from a natural rubber was utilized as a simple model; (**b**) 2D images of the model of the letter C obtained by applying the CPMG-CP sequence. The reconstructed image almost shows the structure of the model.

A simple rubber model was used to test the spatial resolution of 2D image. A letter C cropped from a natural rubber with *T*_1_ at approximately 20 ms was utilized as shown in Figure 7a. The CPMG-CP sequence for multi-echo acquisition was utilized to obtain a 2D image of the simple model. The letter was a 4-mm thick slice and positioned above the RF sensor. The gradient pulse and echo time T_ED_ for the CPMG-CP sequence in Figure 5b were 0.27 and 0.11 ms, respectively. The *k* space was sampled from negative to positive values along the *x*- and *y*-directions, with the gradient amplitude increasing in 11 steps in both directions. Approximately 10 mm FoV along both directions was obtained, and the spatial resolution was about 1.0 mm. Phase cycle was utilized for the CPMG-CP sequence to sample both components of the magnetization vector [4]. Thus, only a single scan was required per gradient amplitude. A total of 120 echoes were obtained and averaged for SNR improvement. A 2D image of this model was obtained by employing a recycle delay of 100 ms and 64 scans, and the total experimental time was 26 min. The cross-section of this model was imaged, as shown in Figure 7b. The 2D image almost showed the structure of the letter, and the different spatial localization can be discriminated across the selective slice.

**Figure 8:**
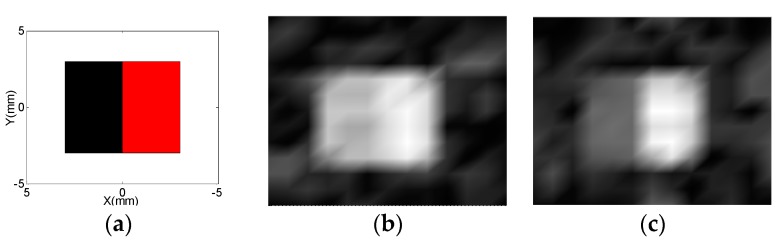
(**a**) Two different kinds of dead pig skin were used to build a burn model. The left half was cut from the abdominal skin of dead pig and used as the control part. The right half was built of the abdominal skin of dead pig immersed in boiling water for 10 seconds to produce full-thickness burns. The T_2_ of the control part was about 19 ms and the T_2_ of the burn part increased to about 35 ms. The images obtained by adding (**b**) the echoes 0–20 and (**c**) the echoes 200–400 show different intensity ratios between the two regions.

Burns are commonly divided into partial-thickness burns that heal by rapid re-epithelialization with conservative therapy, and full-thickness burns that require skin grafting [1]. A simple burn model was built using two kinds of dead pig skin shown in Figure 8a. The left half was cut from the abdominal skin of dead pig and used as the control part. The right half was built of the abdominal skin of dead pig immersed in boiling water for 10 s to produce full-thickness burns [29]. Upon removal from the water, this part of the pig skin was quickly dried. The right half was used as the burn part. Although the burn model was not a standard animal burn model [29] for the model being built after the pig being sacrificed, it would not influence on test results, because the purpose of building this model was to reconstruct the difference of spatially resolved T_2_ image [8] between the control part and the burn part. Moreover, the T_2_ of the control part was about 19 ms and the T_2_ of the burn part increased to about 35 ms. The epidermis and the upper dermis of both parts with the thickness 0.3 mm were tested. The echo time *T_ED_* was set at 0.12 ms in the CPMG-CP sequence, and 1500 echoes were sampled. The gradient amplitude was increased in 11 steps with the FoV at 10 mm. The repetition time was set to 1.0 s, and 32 scans were averaged for SNR improvement. Different T_2_ weighted images were reconstructed from the addition of the echoes shown in Figure 8b, 0–20, and 8c, 200–400. The highest intensity of each image was normalized to one. From Figure 8b, there is only a small difference of the intensity between the two parts, and from Figure 8c a strong contrast of the intensity is visible between the two parts and the intensity of burn part (right half) with the longer T_2_ is much stronger than the control part (left half) with the shorter T_2_. Because proton NMR could distinguish partial thickness burns from full-thickness burns [29], this single-sided MRI probe can be employed to test skin burns.

Combing the 2D phase encoding method with slice selection and a lift system, 3D spatial resolution could be achieved and 3D structure of the skin could be obtained. Because the strong *z*-gradient (7.46 T/m) produces a selective excitation of thin, flat slices of about 300 µm at a distance of 15 mm from the magnet array, the thickness of the imaged slice about 0.3 mm was not enough to excite the whole skin in one shot, a lift system similar to the one in commercial single-sided systems [30] was used and different depths such as the superficial skin or deep skin could be excited with the same resonance frequency. The 3D spatial resolution could be obtained by repositioning of the sensitive slice through the object. This configuration and protocol resulted in a penetration depth of approximately 2.0 mm and it was helpful to quantify the burn depth.

## 4. Conclusions

A new low-field unilateral MRI probe was created with a reduced Halbach magnet, a uniplanar gradient coil system utilizing the mainstream target field method and a uniplanar RF coil using time-harmonic inverse method. Moreover, a simple burn model was 2D imaged using the multiecho accumulation sequence. However, for assessing burn depth in clinical applications, sensitivity requires improvement, penetration depth should be increased, and imaging times should be further reduced. The following methods could be considered to optimize the probe in the future:

(1) The homogeneity of the *B*_0_ field should be improved to improve SNR. Because of the machining tolerance of the aluminum box, a deviation was observed between the measurement and simulation of the *B*_0_ field. Tolerance reduction and performing the shimming work could be considered in our future work.
(2) An instability in the probe was observed. This instability could be attributed to the inconstant temperature because the magnetic field of NdFeB was susceptible to temperature. In the future, we will focus on adding a thermal vacuum insulation layer for the unilateral MRI magnet.
(3) A signal-processing method and a faster imaging method will be considered to accelerate imaging times in unilateral MRI devices in our future work. Nevo’s group [9,31,32] has presented a statistical signal-processing method [31,32] to improve imaging capabilities by improving the extraction of image information from the noisy data, and has also presented modified compressed sensing (CS) and fast spin echo (FSE) methods [9] to accelerate imaging on a unilateral NMR scanner. Therefore, we will consider those methods to accelerate imaging in our unilateral MRI probe.
(4) Additional information such as depth–relaxation map, depth–diffusion map [6] and T_1_ weighted image with a shortened repetition delay [13] will be utilized to assess burn depth. Both depth–relaxation map and depth–diffusion map can be provided by low-gradient unilateral MRI probe. For the low-gradient single-sided NMR sensor, the human skin can be profiled in one-shot. The difference in those maps between different burn degrees will be researched.
